# Case report: Diverse immune responses in advanced pancreatic ductal adenocarcinoma treated with immune checkpoint inhibitor-based conversion therapies

**DOI:** 10.3389/fimmu.2024.1326556

**Published:** 2024-02-13

**Authors:** Xiaoying Li, Chaoxin Xiao, Ruizhen Li, Pei Zhang, Heqi Yang, Dan Cao

**Affiliations:** ^1^ Division of Abdominal Tumor, Department of Medical Oncology, Cancer Center, State Key Laboratory of Biotherapy, West China Hospital, Sichuan University, Chengdu, Sichuan, China; ^2^ State Key Laboratory of Biotherapy and Cancer Center, West China Hospital, Sichuan University, and Collaborative Innovation Center for Biotherapy, Chengdu, Sichuan, China

**Keywords:** pancreatic ductal adenocarcinoma, tumor immune microenvironment, tyrosine kinase inhibitor, immune checkpoint inhibitors, chemotherapy

## Abstract

Pancreatic ductal adenocarcinoma (PDAC) is often diagnosed at an advanced stage, presenting limited therapeutic options and a grim prognosis due to its aggressive nature. Despite ongoing exploration of various combination therapies, a standardized treatment approach after the first-line treatment progress remains elusive. This report details the cases of two patients with unresectable advanced PDAC who underwent distinct conversion treatment regimens involving immune checkpoint inhibitors (ICIs). Remarkably, both patients became eligible for surgery following different anti-PD-1 antibody-based conversion therapies, ultimately achieving R0 resection. In essence, our findings highlight the efficacy of the anti-PD-1 antibody combined with a tyrosine kinase inhibitor (TKI) regimen and chemotherapy alongside anti-PD-1 antibody as viable conversion therapies for preoperative advanced PDAC. Tumor immune microenvironment (TIME) analysis underscores the intratumoral and intertumoral heterogeneity observed in the postoperative immune landscape of surgical specimens. This insight contributes to a deeper understanding of the potential benefits of these conversion therapies in addressing the challenging landscape of advanced PDAC.

## Introduction

Pancreatic ductal adenocarcinoma (PDAC) stands out as one of the deadliest cancer types, impacting around half of diagnosed patients at an advanced stage with a disheartening 5-year survival rate below 9% (1). In recent years, two groundbreaking combination treatments, FOLFIRINOX (5-fluorouracil (5-FU), leucovorin, irinotecan, and oxaliplatin) and GA (gemcitabine with nab-paclitaxel), have emerged as primary therapeutic options for advanced PDAC patients (2, 3). Patients with disease progression on their initial regimen may switch to the alternative combination, or for those not previously treated, a second-line option with liposomal irinotecan and 5-FU exists (4). There are limited treatment options for pancreatic cancer, and the success rate of available treatments is often low (5).

Genomic analyses in PDAC reveal molecular subtypes, the basal-like (or squamous) subtype and the classical subtype, representing the simplest molecular classification (6, 7). PDACs exhibit cellular and spatial biological heterogeneity, characterized by a prominent stromal microenvironment, scarcity of cytotoxic lymphocytes, and hypovascularity (1, 8, 9). This challenging environment, with hypovascularity and vascular compression, contributes to chemotherapy resistance by hindering drug delivery (1, 8, 9). Elevated FAK activity in human PDAC tissues correlates with fibrosis and poor CD8(+) cytotoxic T-cell infiltration. Inhibiting FAK overcomes fibrosis and immunosuppression, rendering tumors responsive to immunotherapy (10).

PDAC is marked by prevalent mutations in KRAS, TP53, SMAD4, and CDKN2A, each mutated in over 50%, although not mutually exclusive (11–13). Despite the prevalence of KRAS mutations (90% in the US, 87% in China) in PDAC, no approved drug targets the KRAS gene (5). DNA damage response (DDR) in patients suggests potential sensitivity to platinum-based and PARP inhibitor treatments (14). Microsatellite instability (MSI), a rare occurrence (1%–2%) in surgically resectable PDACs, poses challenges for ICIs, showing limited efficacy (15, 16). Stereotactic body radiotherapy (SBRT) with nivolumab ± ipilimumab exhibits clinical antitumor activity and manageable safety in refractory metastatic pancreatic cancer (mPC) (17). The contribution of SBRT and how immunotherapy enhances antitumor activity in mPC require further evaluation. Insights into the TIME may inform strategies to address PDAC, where ICIs and targeted therapies have yet to succeed.

Neoadjuvant chemotherapy is being used more frequently in patients with resectable or borderline resectable PDAC, making initially unresectable tumors suitable for surgical intervention. Neoadjuvant therapy is widely employed in treating gastroesophageal cancers and colorectal cancer, demonstrating reduced tumor volume, increased resection rates, and improved survival rates compared with surgery alone (18). Clinical trials explore ICIs-based therapies, such as sintilimab (anti-programmed cell death protein 1 [PD-1]) plus CapeOx (capecitabine + oxaliplatin), showing promising response rates (1-year DFS (90.3%) and OS94.1%) in gastric cancers (19). In PDAC, neoadjuvant gemcitabine-based chemoradiotherapy or chemotherapy demonstrates therapeutic responses, yet standardized guidelines for locally advanced unresectable PDAC are lacking (20, 21), while according to the PREOPANC trial results, there were no overall survival benefits (22). Neoadjuvant therapy can provide the chance of surgical resection for a highly selected cohort of patients with locally advanced PDAC and associated with improvement of overall survival (23). No standardized treatment guideline for locally advanced unresectable PDAC has yet been established. The clinical value of neoadjuvant therapy remains elusive.

In conclusion, our study aims to uncover the posttreatment microenvironment in two patients subjected to different ICI-neoadjuvant therapies. We seek to unveil sensitivity and resistance mechanisms, exploring promising avenues for novel strategies. Our goal is to investigate the potential of personalized multimodal therapy based on individual characteristics.

## Case presentation

### Patient 1

A 56-year-old woman diagnosed with stage IIIB (cT4NxMx) pancreatic ductal adenocarcinoma (PDAC) was deemed unresectable during the initial procedure. After histopathological examination, standard first-line treatment with gemcitabine plus nab-paclitaxel was initiated. Upon progression, the patient faced a bowel obstruction, leading to surgical intervention. Molecular-targeted agents (sulfatinib, a tyrosine kinase inhibitor) combined with tislelizumab (an anti-PD-1 antibody) were subsequently employed. Despite initial non-suitability for surgery, both anti-PD-1 antibody-based conversion regimens enabled R0 resection. Imaging assessments indicated tumor shrinkage after combination therapy, allowing for pancreatectomy. Postsurgery, the patient continued with adjuvant therapy (sulfatinib + tislelizumab).

### Patient 2

A 60-year-old man with cT4NxMx PDAC received gemcitabine + nab-paclitaxel along with immune checkpoint inhibitors (tislelizumab) as first-line treatment. Laparoscopic exploration deemed him unsuitable for surgery initially. However, through an anti-PD-1 antibody-based conversion regimen, R0 resection became achievable. Imaging assessments revealed tumor shrinkage, paving the way for pancreatectomy. Patient 2, upon follow-up, remained alive without recurrence.

### Conversion therapies and surgical intervention

Both patients underwent successful conversion therapies based on immune checkpoint inhibitors (ICIs). Imaging assessments post-conversion therapy displayed tumor shrinkage, making them eligible for surgical intervention. Pancreatectomy was performed, and the patients showed positive outcomes during follow-up (**Table 1**).

**Table 1:** Baseline characteristics before and after surgery.

	Patient 1	Patient 2
**Gender**	Female	Male
**Age**	56	60
**CA-199**	8404 U/ml	600 U/ml
**Intraoperative exploration**	Unresectable	Unresectable
**PD-L1 status**	PD-L1 (TPS: 5%, CPS: 7)	NA
**TNM stage**	cT4NxMx	cT4NxMx
**Treatment schedule**	1:GA 5cycles 2: sulfatinib + tislelizumab (5 cycles) 3: surgery	1:GA + tislelizumab (4 cycles) 2: surgery
**Efficacy evaluation**	PD (progression disease)	PR (partial response)
**Gene status**	dMMR, KRAS p.G12D mutation, ARID1A mutation, TMB22.00mutations/mb	Unknown
**Postoperative staging**	pT2N1MO	pT4N1M0
**Tumor regression**	TRG 2	TRG 2

Annotation: TPS, tumor cell proportion score; CPS, combined positive score; GA, gemcitabine plus nab-paclitaxel; TMB, tumor mutational burden; dMMR, deficient mismatch repair; TRG, tumor regression grade; NA, not available.

## Immune characteristic of surgical specimens

Locally advanced PDAC poses a challenge for surgical resection. The increasing use of neoadjuvant chemotherapy in initially unresectable cases has expanded our surgical options, yet the impact on the TIME remains poorly understood. In order to better investigate the TIME after combination therapy in patients, we performed multifluorescent immunohistochemistry (mfIHC) detection on the patients to visualize how exposure to immunotherapy can reshape the TIME.

**Figure 1:**
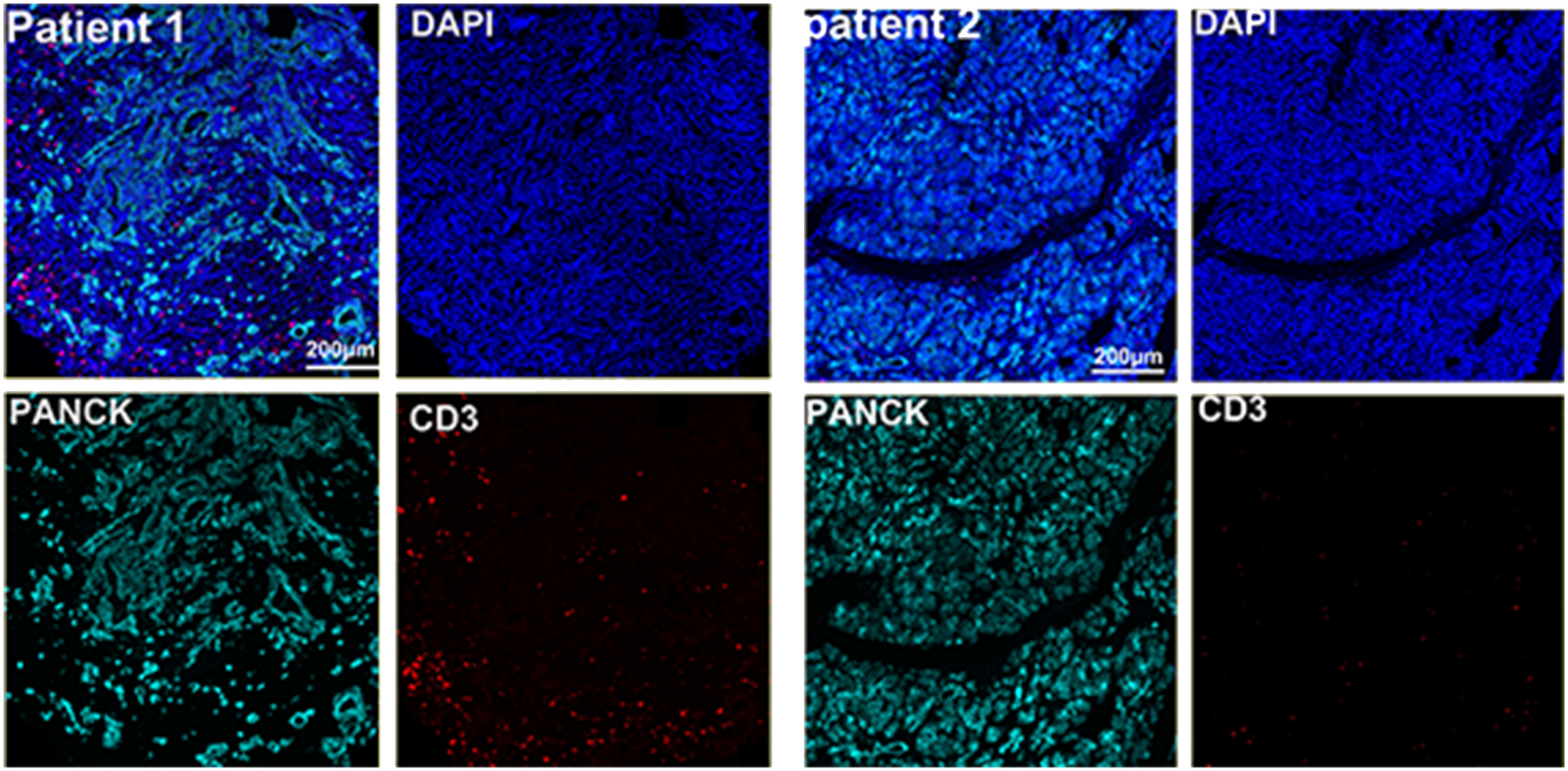
Preoperative immune status of patient 1and immune infiltration in adjacent normal tissue of patient 2.

**Figure 2:**
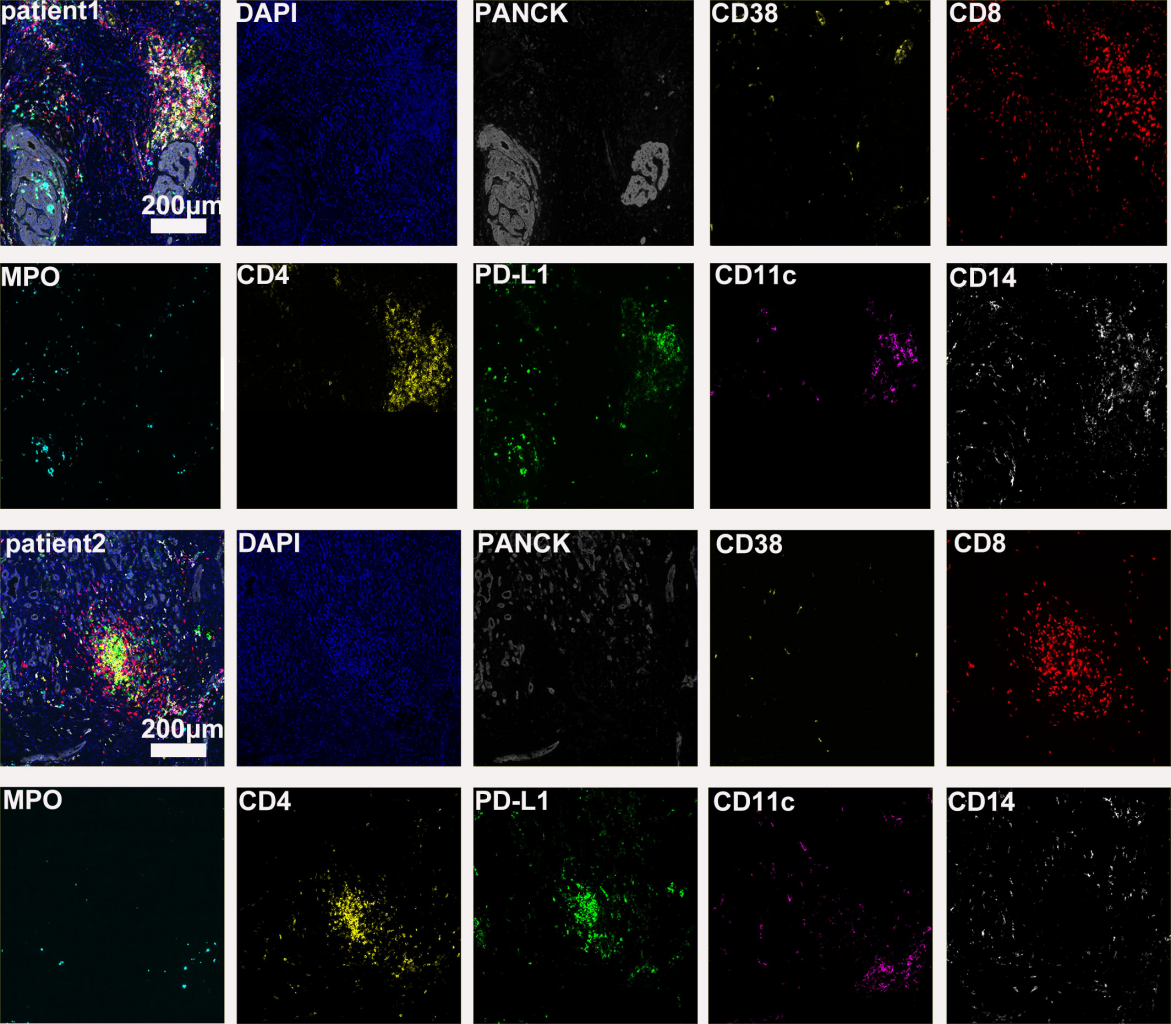
Further postoperative immune status of both patients (panel 2: DAPI, PANCK, CD38, CD8, CD8, MPO, CD4, PD-L1, CD11c, CD14).

**Figure 3:**
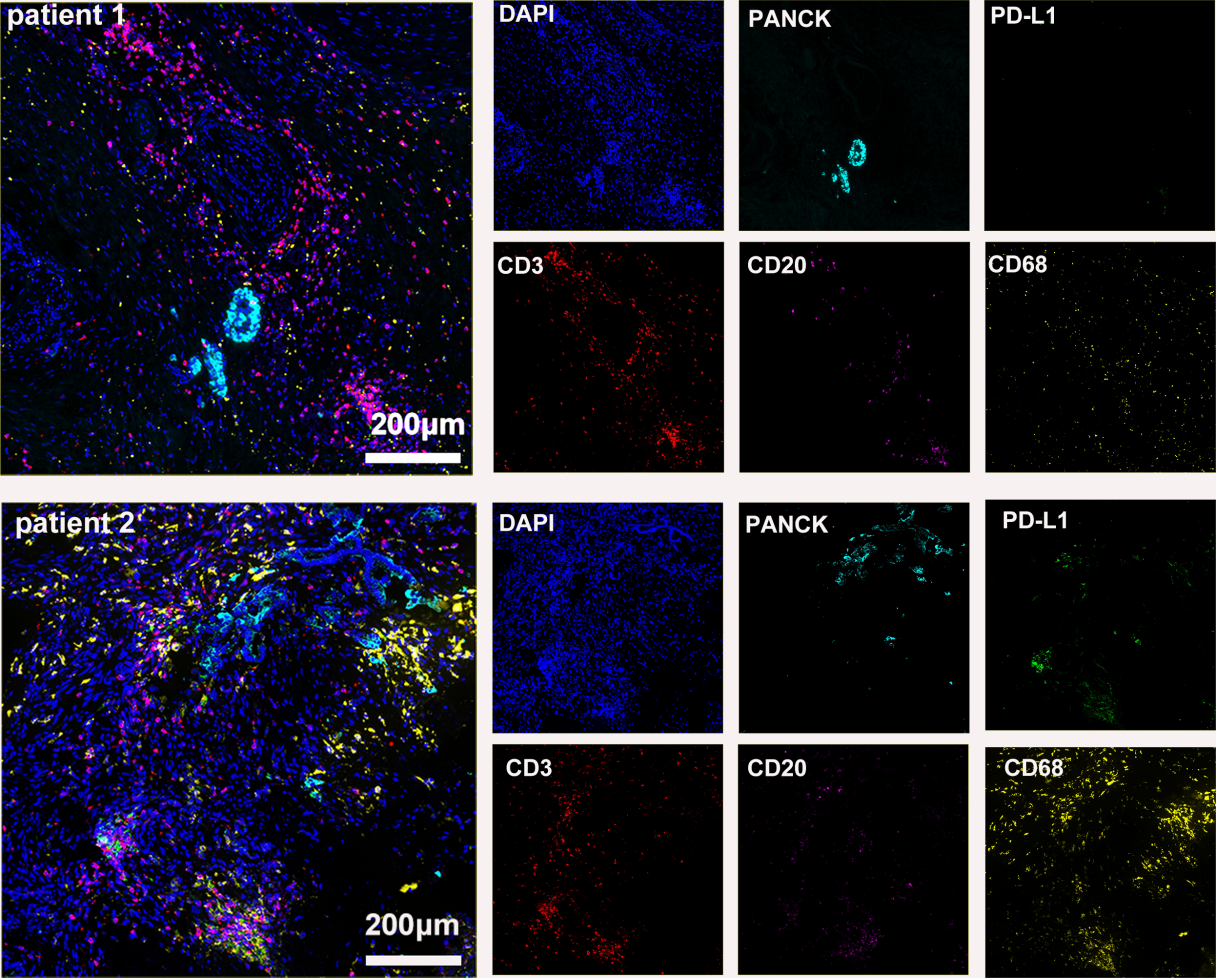
Basic immune characteristics of postoperative specimens (panel 1: DAPI, PANCK, PD-L1, CD3, CD20, CD68).

**Figure 4:**
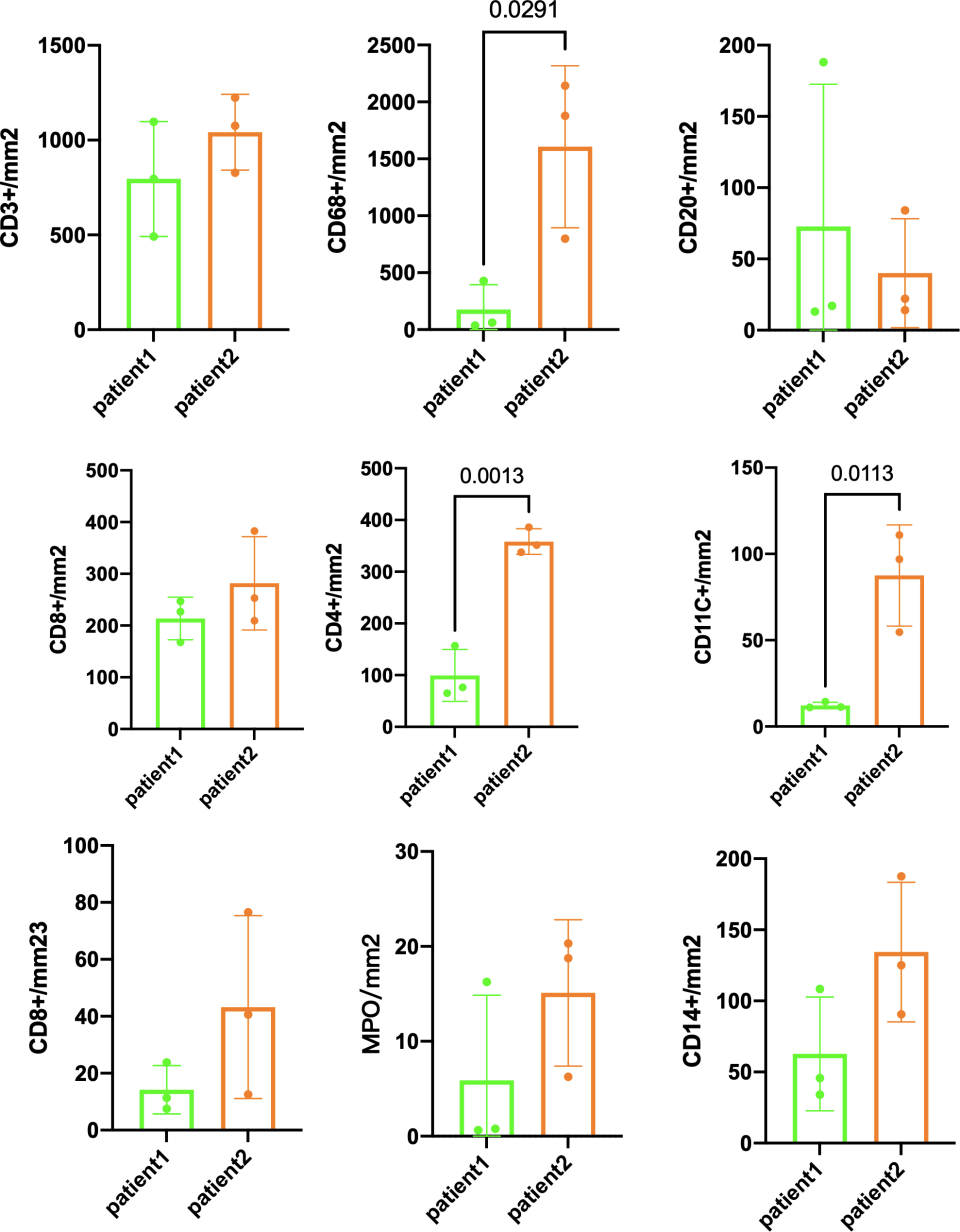
Immune infiltration comparison postsurgery between patient 1 and patient 2. Three randomly selected tumor areas of 1 mm^2^ each were analyzed.

The preoperative biopsy specimen of patient 1 with dMMR suggests CD3+T-cell aggregation at the tumor’s periphery rather than the center (**Figure 1**). After experiencing the failure of first-line GA regimen treatment, the use of sulfatinib + tislelizumab treatment provided an opportunity for surgery. In patient 1, there is a higher prevalence and proportion of CD20+B-cell aggregation (**Figure 2**). Moreover, after receiving treatment with immune checkpoint inhibitors, patients show a trend of increased T cells and B cells. Furthermore, compared with preoperative and postoperative specimens, there is a higher accumulation of lymphocytes in the tumor center than at the tumor periphery. Patient 2 had a biopsy performed at another hospital, so the limited sample size prevented the acquisition of preoperative specimens. Patient 2, after undergoing 4 cycles of GA + tislelizumab, also gained the opportunity for surgery. Postoperative specimens indicate limited CD3+T lymphocyte infiltration in the adjacent normal tissue surrounding the tumor (**Figure 1**). While more lymphocytes were seen in the core of tumor center (**Figure 3**). After undergoing chemotherapy and combination immunotherapy, patient 2 exhibited a significantly higher density of CD68+macrophages, CD4 + T lymphocytes, and CD11c+ dendritic cells compared with patient 1 (**Figure 4**).

In randomly selecting three equally sized regions of interest for comparison in two patients, we observe a trend of immune cell clustering in the treated tumors, particularly in areas centered around PD-L1 expression (**Figures 2**, **3**). Notably, CD68-positive macrophages, CD4+T lymphocytes, and CD11c +dendritic cells show a relative increase in patient 2 (**Figure 4**). The presence of M1-polarized macrophages alongside tumor cells is correlated with improved outcomes in patients who received neoadjuvant treatment (24). Despite residual tumors after immune checkpoint inhibitor (ICI) treatment, both patients exhibited notable enrichment of immune cells, showing extensive intratumoral and intertumoral heterogeneity in the immune landscape. While no significant differences were observed in other cell types, these findings may suggest an activation of the immune state following treatment and residual tumor lesions possess efficient immune response (**Figure 4**).

## Discussion

The expanding application of immune checkpoint inhibitors (ICIs) in perioperative settings, especially in microsatellite instability-high (MSI-H) and/or mismatch repair-deficient (dMMR) rectal/gastric cancers, reflects a promising trend (25–27). Over the last few years, there has not been much success in improving the treatment of pancreatic cancer through monotherapy or immunotherapy. ICIs only demonstrate effectiveness and good tolerability in individuals with advanced pancreatic ductal adenocarcinoma (PDAC) characterized by microsatellite instability (MSI) or dMMR (28, 29). Pancreatic ductal adenocarcinoma exhibits limited responses to immunotherapies, posing challenges in identifying predictive markers for such responses. In contrast to other tumors, there is no established link between PD-L1 expression and the response to PD-1-targeted immunotherapies in pancreatic ductal adenocarcinoma patients (30). The lack of adequate time for the development of a robust adaptive immune response could be a contributing factor to the ineffectiveness of immunotherapies in patients with advanced pancreatic ductal adenocarcinoma (31). Novel approaches for identifying pancreatic ductal adenocarcinoma patients who could derive benefits from immunotherapies may be formulated by leveraging our enhanced comprehension of the immune landscape and its regulator before integration into the neoadjuvant context (32). It is conceivable that characterizing which patients derive clinical benefit from a certain neoadjuvant treatment would be very valuable in identifying to specific patients.

Pancreatic cancer creates a diverse and immunosuppressive environment, which may affect how well immunotherapy works. Infiltration of cytotoxic T lymphocytes (CTLs) is vital for active tumor immunity (33). Tumor-infiltrating T cells (CD3+, CD4+, and CD8+) cells have been associated with improved survival outcomes in individuals with pancreatic ductal adenocarcinoma, especially when these T cells are in close proximity to the tumor cells. Notably, a heightened concentration of CD3+ T cells, especially in close proximity to tumor cells, emerges as a significant predictor for a prolonged progression-free survival period (34). Current trends suggest higher CTLs in MSI patients compared with microsatellite-stable (MSS) patients (35). In gastric cancer, the infiltration of CTLs is greater in the intestinal subtype, regardless of MMR status (36). Further investigations are needed to understand the relationship between MSI status and immune cell infiltration within the tumor.

Neoadjuvant therapies have been reported to reshape the cancer microenvironment and convert the immunosuppressive behavior into an anti-tumorigenic state, such as facilitate infiltration with natural killer cells and CD4+ T cells as well as decreased stromal activation (24). For instance, in gastric cancer, through comparing the transcriptional profiles of TME pretreatment and posttreatment, implicating that cancer cells can trigger vascularity by stromal cell remodeling (37). Neoadjuvant chemotherapy-treated tumors were inclined to have a T-cell-rich pattern (higher CD8:CD4 ratio) with reduced immunosuppressive granulocytes and macrophages; in addition, after neoadjuvant chemotherapy, the spatial location of M1-polarized macrophages was closer to tumor cells and indicate higher pCR and better survival (38). Therefore, multidimensional omics analysis approaches like single-cell analytics applied in well-designed clinical trials may aid addressing post-therapy cellular plasticity and TME remodeling. Incorporating targeted therapies into the neoadjuvant context would necessitate prior molecular analysis of tumor material; however, current surrogate markers for assessing neoadjuvant therapy response lack reliability. In our study, we demonstrate that the potential of a combined tyrosine kinase inhibitor (TKI) and anti-PD-1 antibody can effectively slow down the progression of PDAC. Additionally, treating unresectable PDAC with gemcitabine + nab-paclitaxel combined with immune checkpoint inhibitors (tislelizumab) as the initial approach creates a surgical opportunity for unresectable PDAC. While Patient 1, diagnosed with dMMR status and resistant to standard treatment, exhibited a positive response to neoadjuvant ICI-based therapies, ultimately facilitating subsequent surgery. However, the additive effect of TKI drugs remains unclear. To better comprehend its impact on the tumor immune environment (TIME), we prompt investigations into postoperative specimens, utilizing multifluorescent immunohistochemistry (mfIHC) to visualize the transformative effects of immunotherapy on the TIME. Both patients experienced tumor shrinkage posttreatment, facilitating surgical intervention. Notable intratumor differences were observed between the patients, emphasizing the impact of preoperative treatment on the TIME. Evidence underscores the critical role of the TIME in immunotherapy effectiveness, with preoperative treatment inducing TIME changes (38). However, the prognostic value of immune cell subpopulations within the tumor and its surrounding stroma in immunotherapy response for PDAC remains unclear.

Hence, much of the current research is dedicated to exploring combined therapeutic approaches. These approaches aim to boost T-cell infiltration, improve antigen exposure, enhance antigen presentation, and investigate new potential targets. However, achieving immunotherapy for pancreatic cancer is still a challenging journey. In the neoadjuvant stage, there is a risk of tumor progression until reliable predictive factors are identified. While this approach has shown promise in other cancer types, using checkpoint inhibitors alone or with standard chemotherapy as the first-line treatment for advanced pancreatic ductal adenocarcinoma proved ineffective (30). In line with recent literature, several studies highlight the evolving landscape of immunotherapy in PDAC. Noteworthy advances include investigations into the role of CD40 agonistic monoclonal antibody adoptive T-cell therapies and vaccine-based approaches (39–42). Ongoing clinical trials are actively exploring novel immunotherapeutic strategies for PDAC. Trials investigating combination therapies, such as immune checkpoint inhibitors in conjunction with chemotherapy or targeted agents, are particularly prevalent. Additionally, personalized approaches, including neoantigen-based vaccines and adoptive cell therapies, are being rigorously evaluated. The outcomes of these trials are anticipated to provide valuable insights into optimizing treatment strategies for PDAC.

In conclusion, our findings suggest that immune checkpoint inhibitor-based conversion therapies may influence the immune landscape in locally advanced PDAC, and we observed a trend of immune cell clustering in the treated tumors, particularly in areas centered around PD-L1 expression. The above findings highlight the potential of combination therapies to reshape the TIME and long-term outcomes in challenging cases. A favorable immune microenvironment is often linked to improved survival outcomes and reduced risk of recurrence. Therefore, refractory pancreatic cancer demands advanced multidisciplinary collaboration and comprehensive treatment. The key challenge lies in making immunotherapy more precise to benefit more patients sustainably. PD-L1 expression alone is insufficient, and employing mfIHC with spatial analysis may offer a more effective approach to identify suitable candidates. Future research should explore the relationship between microsatellite instability (MSI) status and immune cell infiltration in PDAC, revealing insights into tumor immunogenicity and responsiveness to immunotherapies. Additionally, investigating immune cell heterogeneity and its correlation with clinical outcomes may lead to robust biomarker identification. Longitudinal studies monitoring changes in the tumor immune microenvironment during treatment are crucial for developing adaptive treatment approaches. Understanding how chemotherapy modulates the tumor microenvironment may optimize combination therapies with immunotherapeutic agents. Lastly, studying the interplay between immunotherapy and conventional treatments is essential for refining combination strategies and improving treatment efficacy.

## Methods

The evaluation of PDAC in human specimens entailed the scrutiny of formalin-fixed and paraffin-embedded (FFPE) tissue samples, which were sectioned into 4-μm slices. Each specimen underwent staining with hematoxylin and eosin (H&E). To prepare for immunohistochemistry (IHC) and multiplex immunohistochemistry (mIHC), the tissue sections underwent deparaffinization using xylene, followed by rehydration through a series of graded ethanol solutions (100%, 95%, and 70%). Subsequently, the slides underwent microwave treatment, and antigen retrieval was facilitated through a 15-min exposure to EDTA Target Retrieval Solution pH 9.0 (ZLI-9068).

For mIHC analysis of human samples, primary antibodies such as PD-L1 (HUABIO; HA721176; 1:1,000), CD3 (HUABIO; HA720082; 1:1,000), CD4 (HUABIO; ET1609-52; 1:1,000), CD68 (HUABIO; HA601115; 1:3,000), CD8 (ImmunoWay; ABT304; 1:200), CD11c (HUABIO; ET1606-19; 1:1,000), CD38 (HUABIO; CD38; 1:1,000), CD20 (HUABIO; HA721138; 1:1,000), MPO (Abcam; ab208670; 1:1,000), and pan-cytokeratin (HUABIO; HA601138; 1:3,000) were utilized. Subsequently, the slides underwent a 20-min incubation at room temperature with secondary antibodies (HRP Conjugated Goat anti-Rabbit IgG: HA1119 or HRP Conjugated Goat anti-Mouse IgG: HA1120). Removal of all antibodies, including primary and secondary antibodies, was conducted according to the instructions provided by LuminIris (MH010101). The reagents and techniques employed in whole-mount immunostaining were sourced from the IRISKit HyperView mIF kit. Multispectral images were scanned with VS200, and QuPath was employed for mIHC analysis (43).

## Data availability statement

The original contributions presented in the study are included in the article/supplementary materials, further inquiries can be directed to the corresponding author/s.

## Ethics statement

The studies involving humans were approved by bioethics review board of West China Hospital, Sichuan University. The studies were conducted in accordance with the local legislation and institutional requirements. The participants provided their written informed consent to participate in this study. Written informed consent was obtained from the individual(s) for the publication of any potentially identifiable images or data included in this article.

## Author contributions

XL: Writing – original draft. CX: Writing – review & editing. RL: Writing – review & editing. PZ: Writing – review & editing. HY: Writing – review & editing. DC: Writing – original draft.
